# Alloying metal cations in perovskite nanocrystals is a new route to controlling hot carrier cooling

**DOI:** 10.1038/s41377-023-01316-x

**Published:** 2023-11-20

**Authors:** Navendu Mondal, Ben P. Carwithen, Artem A. Bakulin

**Affiliations:** Department of Chemistry and Centre for Processable Electronics, Imperial College London, London, W12 0BZ UK

**Keywords:** Ultrafast photonics, Nanoparticles, Optical spectroscopy

## Abstract

Hot carrier cooling is slowed down upon alloying tin in lead-halide perovskite nanocrystals through the engineering of carrier-phonon and carrier-defect interactions.

## Abstract (web summary)

The key electronic and phononic processes governing hot carrier cooling dynamics in perovskite nanosystems can be tuned through Pb-Sn alloying.

## Graphical abstract

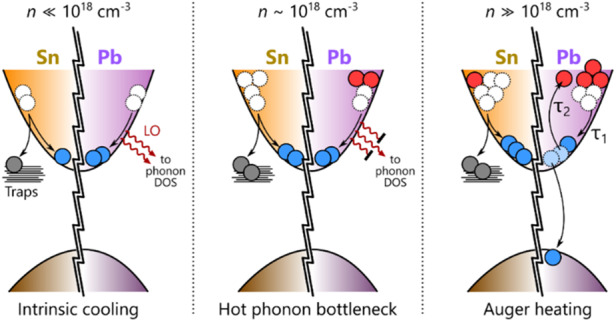

In semiconductors, incident photons possessing energies equal to or greater than the bandgap transit electrons between the valence and conduction bands, leading to the formation of mobile free carriers. Under above-gap excitation, the ‘excess’ energy also breaks the thermal equilibrium between the free carriers and the lattice to create a new transient distribution of ‘hot’ electrons and holes in their respective bands. Hot carriers initially have a higher average energy (or temperature, if transient equilibrium is achieved) than the lattice, but this is quickly dissipated as heat into the surrounding medium. In any type of photovoltaic device, these heat losses result in lower power conversion efficiency. As such, circumventing or slowing this loss pathway is one approach towards achieving higher device efficiency. Widely discussed methods of minimising carrier relaxation losses include the development of hot carrier solar cells, and the use of carrier multiplication in semiconductor quantum dots. Successful realisations of either approach could potentially break the theoretical limit for semiconductor photovoltaics^1–3^.

Recently, lead-halide perovskites (APbX_3_) have emerged as processable materials with the potential to outperform conventional semiconductors in photovoltaics due to their exceptional properties such as slow recombination rate, defect tolerance, and long carrier diffusion lengths. In addition, several early studies^4–7^ have revealed various photophysical mechanisms that control hot carrier cooling (HCC) in perovskites, generating excitement and sparking broad discussions about the prospect of hot carrier perovskite solar cells.

Despite the remarkable progress in perovskite photovoltaics in recent years, practical development of hot-carrier perovskite solar cells remains tantalisingly out of reach^1,2^. In the last few years, the research community has made several leaps towards understanding the mechanism of HCC in perovskites^2–9^, from identifying intrinsic factors that could slow down this process to exploring the effect of material morphology, composition, and dimensionality^10–20^. In the composition space, the variation of material properties in response to A-cation and X-halide doping has been studied^10–12,19^. Now, writing in *Light, Science & Applications*, Dai et al. report evidence of slower HCC upon increasing the Sn content in Pb-Sn alloyed perovskite nanocrystal systems^18^.

The overall HCC mechanism can be divided into a series of steps in any perovskite system, including the one studied by Dai et al.^18^ (Fig. 1). First, the absorbed photons generate free electrons and holes with a non-equilibrium distribution of excess energies. These charge carriers undergo thermalisation through rapid (10–100 fs) carrier-carrier scattering events and establish an equilibrium distribution characterised by an effective hot carrier temperature. These thermalised hot carriers exchange energy with the lattice on a 0.1–1ps timescale via carrier-phonon interactions and eventually cool down to the lattice temperature. Owing to the polar nature of perovskite semiconductors, the dominant pathway of relaxation is considered to be the Fröhlich interaction between hot carriers and longitudinal optical (LO) phonons, which ceases once the excess energy of the hot carriers falls below that of the LO phonon energy gap. Thereafter, the emitted LO phonons decay into daughter longitudinal acoustic phonon branches (through Klemens pathway), which spread the heat across the material (or device) on the macroscale, depending on the thermal conductivity of the system. As evident from the mechanism indicated above, a viable route towards slowing down HCC could be in carefully blocking one or more of the intermediate energy dissipation steps.

**Fig. 1:**
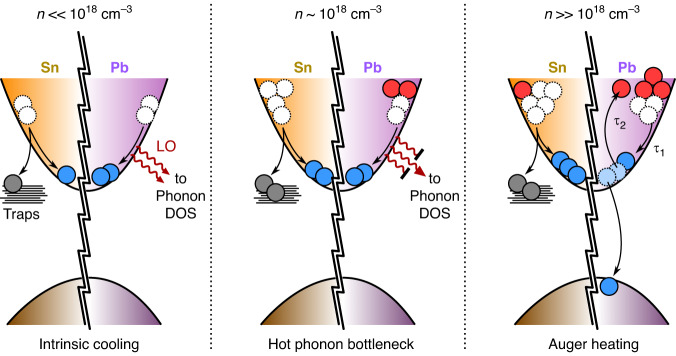
Hot carrier cooling mechanisms in perovskites, including Pb-Sn alloy NCs. At low-to-moderate carrier density, HCC is limited by trapping and the hot phonon bottleneck effect; while Auger heating dominates at high carrier density (DOS = density of states)

As the first stage of HCC is mainly governed by their coupling to LO phonons derived from the Pb-X sub-lattice, one would expect similar HCC dynamics across the lead-halide perovskite family. However, differences in HCC have been observed for hybrid (A = methylammonium, formamidinium) perovskites compared to their all-inorganic Cs-based counterparts and polaron formation, thought to depend on the nature of the A-cation, has been suggested as a possible explanation for this^6^. In perovskite systems, HCC can also be slowed by the hot phonon bottleneck (HPB) effect that becomes prominent under high carrier densities. Conceptually, this can be regarded as the increased competition for a finite availability of cold LO phonons into which the carriers may deposit their excess energy^4–13^. Furthermore, acoustic-optical phonon upconversion could also contribute to the slowing down of HCC, as reported by Yang et al.^8^.

HCC in perovskite materials, particularly the quantum-confined nanoscale systems, may also be affected by Auger recombination (at high carrier density, usually ~10^19^ cm^−3^), wherein the energy released under the recombination of one electron-hole pair is transferred to a nearby third carrier. This leads to the generation of further hot carriers (known as ‘Auger heating’), opening up another route to sustaining their population over longer timescales^4,19^.

Apart from the major influence of phonons and carrier-carrier interactions, the role of intra-bandgap electronic states such as surface traps or interstitial defects on HCC dynamics remains debated and underexplored^11,18,21,22^. For any given system, small variations in the reported hot carrier lifetime are perhaps due to the lack of proper estimation of the traps present in the system.

This intuitively points towards the identification of each mechanistic route for HCC, and ways to control them require intense research in perovskite composition space. While there is great debate over whether partial or complete replacement of Pb by the non-toxic Sn could offer similar optoelectronic properties, including HCC, rapid oxidation of Sn^2+^ to Sn^4+^ introduces another challenge to the stability of this material. In prior work, Dai & colleagues employed stable pure Sn-based NCs as a testbed to study hot carrier dynamics^17^, and now show that Sn-Pb alloying provides an additional dimension to control HCC^18^.

In this work^18^, Dai et al. introduced Sn in MA and Cs-based lead iodide perovskite nanocrystals and followed the conventional procedure of extracting HCC dynamics from transient absorption spectra^4,5,7,9^. Based on the time-dependent spectral narrowing of the band-edge bleach they tracked the dynamics of hot-carrier temperature within the first 100 ps after photoexcitation. The decay of hot carrier temperature from 1000s to 100s K could be described by a single exponential function at carrier densities lower than ~10^18^ cm^−3^, becoming bi-exponential at >10^18^ cm^−3^. The dominant sub-ps decay (τ_1_) was assigned to carrier-LO phonon coupling, while the secondary few-ps time component (τ_2_) was related to the HPB effect. The researchers observed that τ_1_ and τ_2_ increased with increasing content of Sn in alloyed NCs. Replacement of Pb by Sn increased the material’s dielectric constant, leading to phonon screening, which in turn slowed the τ_1_ decay. Based on previous reports, Dai et al. proposed that the collective contributions of suppressed Klemens decay (due to a greater optical-acoustic phonon energy gap), as well as lower thermal conductivity, slow down τ_2_ with increasing Sn content^18^.

Dai et al. further revealed that shorter τ_1_ and τ_2_ for all the Pb-Sn alloy and pure Sn-based NCs compared to the pure Pb-based NCs are due to the influence of the competing hot carrier trapping process in the Sn-based systems. Indeed, these former systems possess lower photoluminescence quantum yields and shorter band-edge carrier lifetimes due to the abundance of trapping sites. Interestingly, fully inorganic (CsSn_x_Pb_1-x_Br_3_) NCs exhibited an even shorter hot carrier lifetime compared to their hybrid counterpart, possibly due to the presence of more trapping centres in the all-inorganic systems. This is further corroborated by the slowing down of τ_1_ and τ_2_, and higher hot carrier temperature when trap states were passivated via Na-doping^18^.

The study by Dai et al.^18^ opens space for reflection and consolidation of recent developments in the field of HCC in perovskite materials. As far as the HCC mechanism is concerned, it still remains unclear why the nanoscale counterparts of Pb-Sn alloyed systems demonstrate reduced HPB compared to bulk analogues^11^. The answer may lie in the nature of the traps, and it would be of immediate interest to identify and characterise the origin of electronic defects contributing to the acceleration of HCC dynamics. Importantly, it would be also of interest to clarify whether hot carrier trapping can significantly compete with the slowing route of HPB.

Due care also needs to be taken in rationalising and disentangling the role of the HPB from Auger heating effects. Two-pulse (‘pump-probe’) spectroscopic techniques do not provide independent control of the hot and cold carrier sub-populations, and in this regard, complementary three-pulse (‘pump-push-probe’) spectroscopic approaches might appear very helpful^11,16,23^. This knowledge would further facilitate the development of design principles towards slowing HCC at excitation intensities comparable to solar illumination.

Finally, while a respectable decrease in HCC rate is achieved, reports on directly harvesting these short-lived hot carriers remain limited^23,24^. We hope that further studies will open up the path towards applying the concepts discussed above in the practical development of hot carrier solar cells.
